# MICA Expression Is Regulated by Cell Adhesion and Contact in a FAK/Src-Dependent Manner

**DOI:** 10.3389/fimmu.2016.00687

**Published:** 2017-01-19

**Authors:** Gerald Moncayo, Da Lin, Michael T. McCarthy, Aleksandra A. Watson, Christopher A. O’Callaghan

**Affiliations:** ^1^Henry Wellcome Building for Molecular Physiology, University of Oxford, Oxford, UK

**Keywords:** MICA, NKG2D, FAK/Src, adhesion, contact, metastasis

## Abstract

MICA is a major ligand for the NKG2D immune receptor, which plays a key role in activating natural killer (NK) cells and cytotoxic T cells. We analyzed NKG2D ligand expression on a range of cell types and could demonstrate that MICA expression levels were closely linked to cellular growth mode. While the expression of other NKG2D ligands was largely independent of cell growth mode, MICA expression was mainly found on cells cultured as adherent cells. In addition, MICA surface expression was reduced through increase in cell–cell contact or loss of cell–matrix adherence. Furthermore, we found that the reduction in MICA expression was modulated by focal adhesion kinase (FAK)/Src signaling and associated with increased susceptibility to NK cell-mediated killing. While the mechanisms of tumor immune evasion are not fully understood, the reduction of MICA expression following loss of attachment poises a potential way by which metastasizing tumor cells avoid immune detection. The role of FAK/Src in this process indicates a potential therapeutic approach to modulate MICA expression and immune recognition of tumor cells during metastasis.

## Bullet Point Summary

1. MICA was predominantly expressed on adherent cell lines, and this expression was reduced in the absence of cell-surface adherence.
2. MICA downregulation with loss of adherence was modulated through FAK/Src.
3. Cell–cell contact, influenced by extracellular calcium availability, regulated MICA expression.
4. Cellular growth mode modulated NK cell-mediated cytotoxicity, therefore affecting NK cell recognition of metastatic cells.

## Introduction

Tumors are complex networks of cells that can interact with the extracellular matrix and neighboring normal tissues (1). Alterations in cell–cell and cell–matrix interactions are classic phenomena that occur during cancer development and affect disease progression (2). As adherent cancer-derived cell lines lose contact with their underlying surface, they tend to aggregate into three-dimensional tumor-like masses that recapitulate some features of *in vivo* tumor growth (3). These “spheroids” reflect aspects of solid tumors in morphology, compact organization, growth dynamics, capacity to develop a necrotic core, proliferation in the periphery, induction of hypoxia, and increased resistance to chemo- and radiotherapy (4, 5). Spheroids are therefore useful *in vitro* models of cancers for a range of studies (4).

Integrins are major cell–matrix adhesion receptors (6). During adhesion and migration, integrins activate a range of signal transduction molecules, such as focal adhesion kinase (FAK) and the Rous sarcoma oncogene family (Src) (6, 7). FAK and Src signal through PI3K/Akt(PKB)/GSK-3/mTOR (8) and the Ras/Raf-1/ERK pathways (9), and their expression is often deregulated in cancers. Cell–cell adhesion is mediated by proteins including cadherins, immunoglobulin superfamily proteins, EGF family members, C-type lectins, and proteins containing leucine-rich repeats (6, 10). Cell–cell and cell–matrix adhesion receptors participate in intracellular communication linked to the cytoskeleton, affecting cell shape and polarity, cytoplasmic organization, cell motility, intracellular signal transduction, cancer progression, and metastasis (6).

Natural killer (NK), cytotoxic T cells, and gamma-delta T cells are critical cellular effectors of the immune system, which can recognize and kill virus-infected and tumor-transformed cells and can also release chemokines and cytokines, such as tumor necrosis factor-alpha (TNF-α) (11). NK cell activity is modulated by signaling through a complex balance of ligand–receptor interactions (12). Inhibitory receptors recognize a range of ligands including MHC class I molecules and thereby hinder cytotoxicity against normal self-tissues (13).

NKG2D is a key activating receptor expressed on NK cells, cytotoxic T cells, and gamma-delta T cells, which recognizes a variety of ligands including MHC class I-related chain (MIC)-A and -B (14) and the UL16 binding proteins (ULBPs) (15). A wide range of stresses have been shown to modulate the expression of these ligands, including viral infection, oxidative stress (16), heat shock (17), TNF-α (18), metalloproteases that regulate the release of the soluble forms (19), DNA damage, and cell cycle modulators (20). The surface expression of these ligands must be closely regulated to avoid an inappropriate immune assault on otherwise healthy cells. Conversely, if tumors or transformed cells do not express these ligands, this will facilitate their escape from recognition.

This study demonstrates that MICA, a key activating ligand for NKG2D, is mainly expressed on adherent cells and that this expression is reduced upon loss of surface attachment and increased cell–cell contact, underscoring the importance of the FAK/Src signaling pathway in modulating MICA expression. Reduced MICA expression upon loss of attachment or increased cell–cell contact results in reduced susceptibility to NK cell killing, suggesting a mechanism whereby metastasizing tumor cells may evade immune recognition.

## Results

### MICA Is Mainly Expressed in Adherent Cell Lines

**Figure 1:**
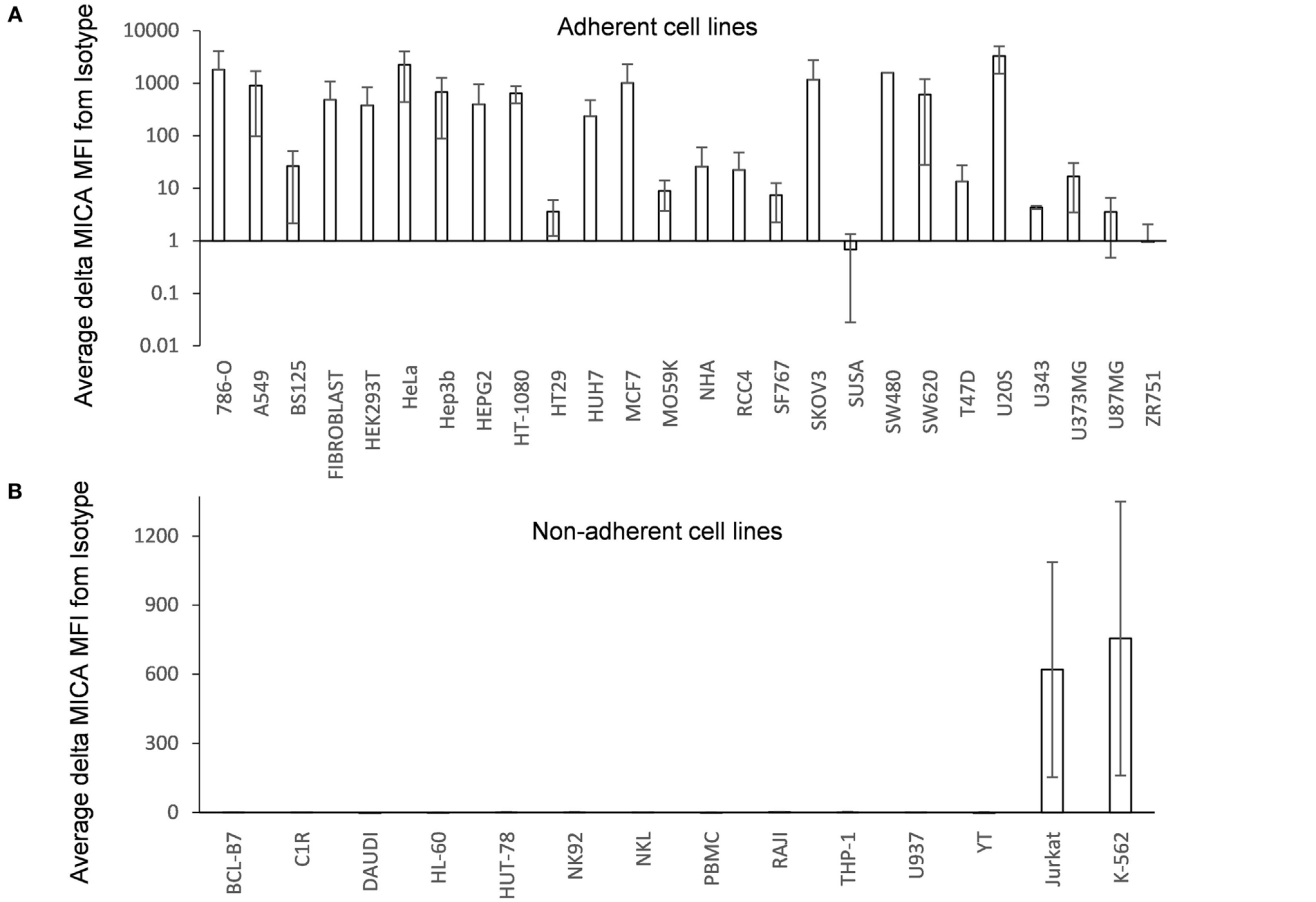

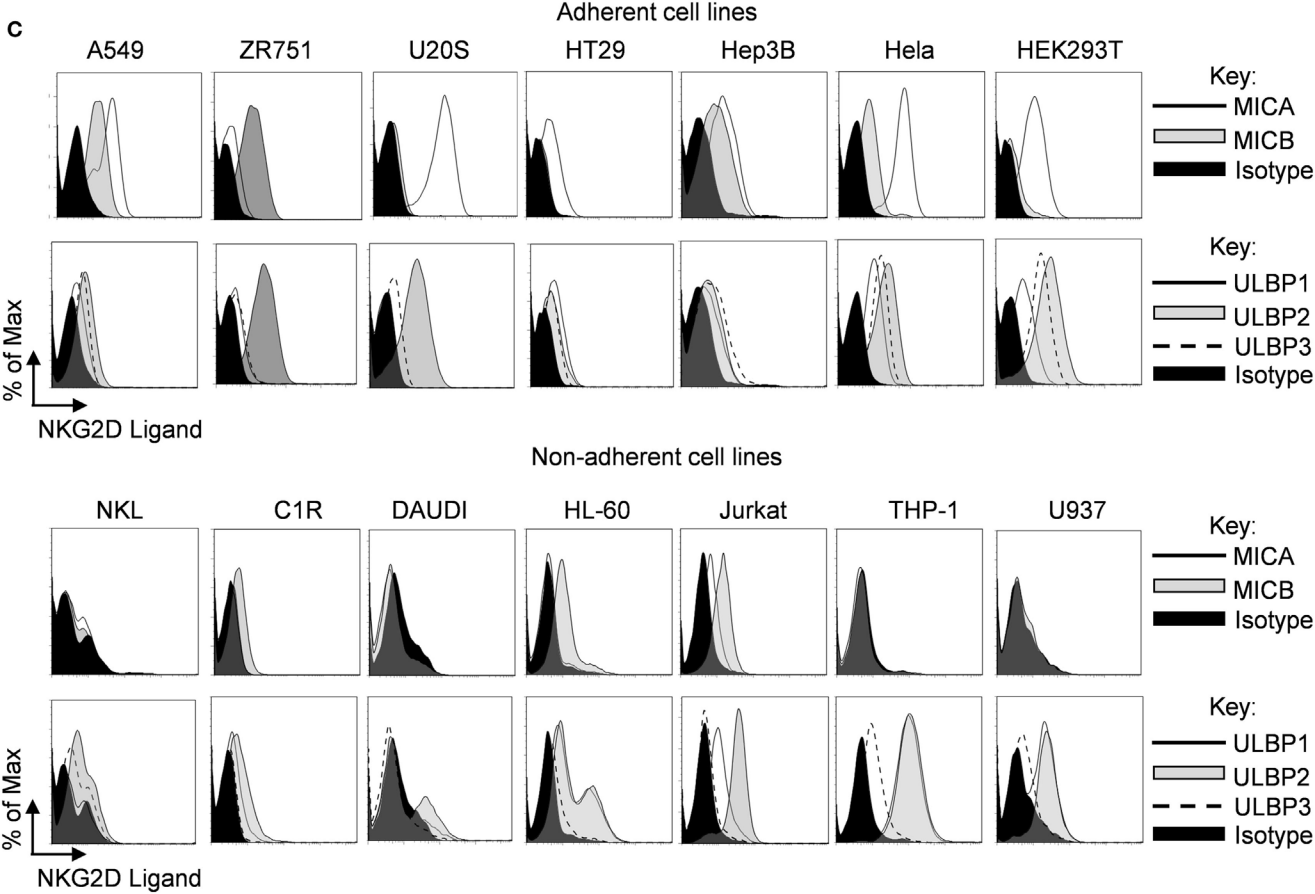
**MICA surface expression on adherent and suspension cells**. **(A)** Adherent and **(B)** non-adherent human cell lines were screened for MICA expression using the anti-MICA monoclonal antibody 2C10. Surface expression was analyzed by flow cytometry. The mean delta MFI as MFI (MICA) − MFI (ISOTYPE) is displayed in the table. Error bars represent the SD of *n* > 3 independent experiments. **(C)** Adherent and suspension cell lines were cultured under normal conditions and stained for MICA or MICB or UL16 binding proteins 1–3 or with an isotype control antibody. Dead cells were excluded using propidium iodide staining. *n* = 3 independent experiments.

A range of human cell lines of different (mainly cancer-derived) origins which were cultured adherently or in suspension were screened by flow cytometry for MICA surface expression. Most adherent cell types tested expressed moderate to high levels of MICA (Figure 1A; Figure S1A in Supplementary Material), including two primary adherent non-cancer cell types growing as monolayers (fibroblasts and normal human astrocytes). In contrast, MICA surface expression was absent or low in most of the suspension cell lines tested (Figure 1B; Figure S1B in Supplementary Material). This was not the case for other NKG2D ligands as ULBPs were often found in suspension cell lines, while MICB was not always expressed in adherent cells (Figure 1C).

### MICA Expression Is Reduced in the Absence of Cell-Surface Adherence

**Figure 2:**
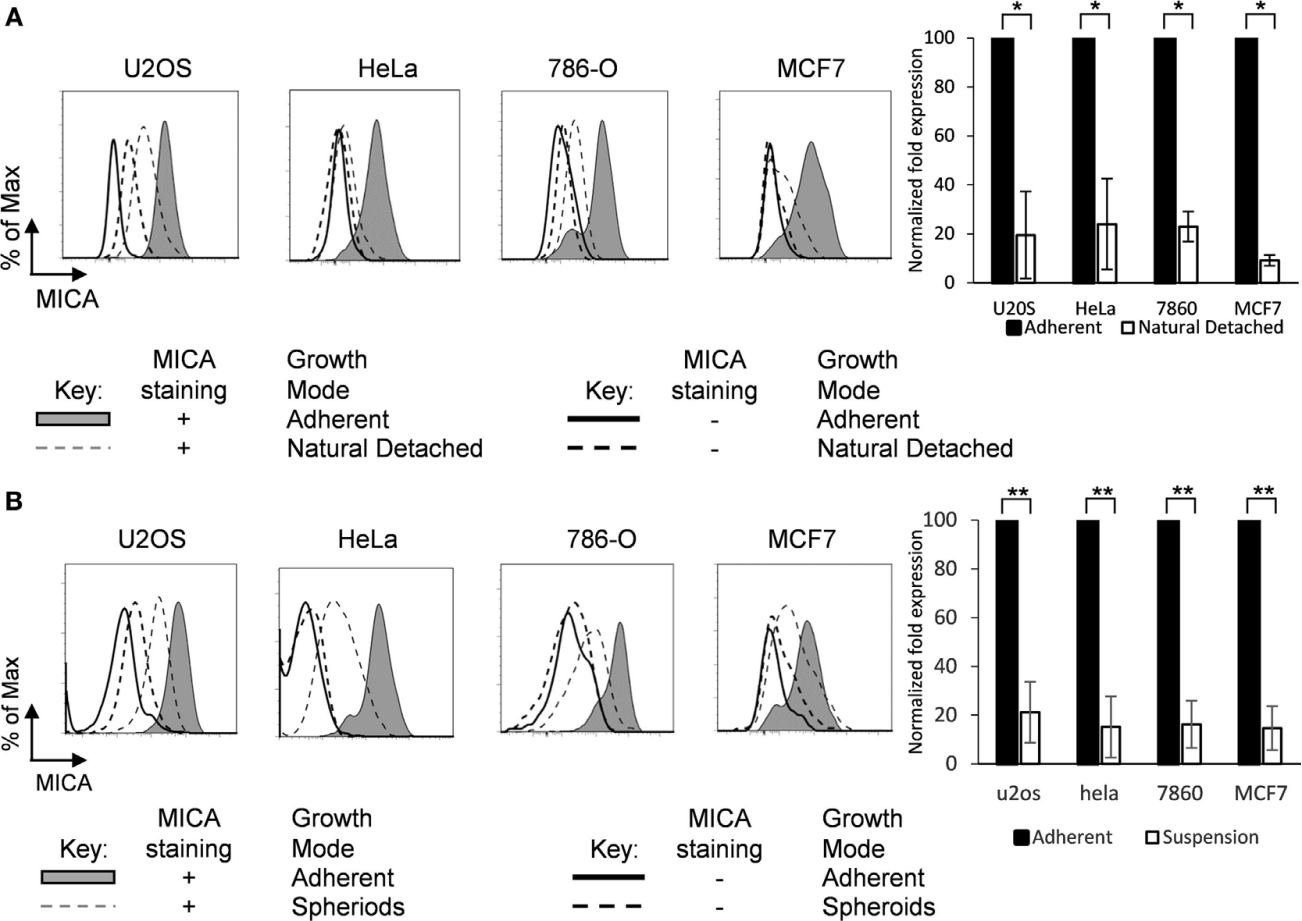
**MICA is expressed on adherent cells and loss of adhesion downregulates surface expression**. **(A)** Adherent cells (shaded gray histogram) and the cells which had detached from the surface within the cultures (“natural detached,” dashed gray line) were stained for MICA or with an isotype control antibody. **(B)** Adherent cell lines were cultured over agarose-coated plates for 5 days to force formation of non-adherent spheroids (dashed gray line) or were cultured under non-confluent adherent conditions (shaded gray histogram) and stained for MICA surface expression (isotype control—black line for adherent cells or dashed black line for non-adherent cells). *n* = 3 independent experiments.

When adherent tumor cell lines are cultured as monolayers, a small proportion of cells detach from the adherent monolayer (Figure S1C in Supplementary Material). To examine whether loss of surface attachment showed a correlation with a reduction in MICA expression, we analyzed MICA expression on adherent cells and their non-adherent counterparts which had detached and were floating in suspension. Up to 60% of cells found in each suspension sample were alive as assessed by propidium iodide staining, and these live cells expressed lower levels of MICA on their surface compared to their adherent counterparts in the same media across a range of cell types (Figure 2A).

Cells which are normally adherent can be forced to grow in suspension by culture over agarose-coated plates, to which they cannot adhere. Under these conditions, they may form multicellular masses called spheroids (Figure S1D in Supplementary Material) (4). To investigate whether loss of surface adhesion would modulate MICA surface expression, cells were cultured as spheroids and MICA expression was compared to the equivalent surface adherent cells. Cells cultured under adherent conditions expressed high levels of surface MICA, but when surface adherence was prevented by culture in agarose-coated plates, MICA expression was reduced substantially (Figure 2B).

### Culture in Suspension Also Alters HLA-A, B, C and MICB, ULBP2, and ULBP3 Expression

As NKG2D has several human ligands, we examined whether growth in suspension affected the surface expression levels of the other NKG2D ligands on U2OS and HeLa cells (Figure S2 in Supplementary Material). Forcing HeLa cells to grow in suspension by culture on agarose resulted in lower levels of expression of ULBP2 and ULBP3 compared to the levels seen on adherent cells, although the effect was less striking than that observed for MICA. MICB and ULBP1 surface expression levels were very low, but even this expression was reduced in suspension cells (Figure S2A in Supplementary Material). As a control, cell-surface expression of the non-NKG2D ligands HLA-A, B, C was assessed on adherent and suspension cultured cells. After growth in suspension, HLA-A, B, C expression was lower than when grown in an adherent fashion but was still very high compared to NKG2D ligands. The same was the case with U2OS cells, which do not express MICB and ULBP1 (Figure S2B in Supplementary Material). In summary, several cell lines expressed lower levels of NKG2D ligands when grown in suspension compared to when cultured as an adherent monolayer.

### MICA Downregulation with Loss of Adherence Is Not Caused by Hypoxia, Apoptosis, or Metalloprotease Action

Hypoxic environments may be found in the center of spheroids (21), but neither hypoxia nor 24 h of hypoxia followed by 24 h of reoxygenation affected MICA surface expression (Figure S3A in Supplementary Material). Similar results were observed following a full hypoxic stimulus with anoxia (0.1% oxygen) in adherent U2OS, HeLa, and 786-O cells (Figure S3B in Supplementary Material). Furthermore, although 786-O cells are deficient in HIF-1a, which is the key transcription factor in the hypoxic response, these cells still demonstrate reduced MICA expression when grown as spheroids compared to when grown as adherent cells. This pattern was unaffected by hypoxia. Therefore, we found no evidence that hypoxia triggered a reduction in MICA expression in adherent cells.

Loss of adherence can induce cell death through mechanisms involving p53, ATM, and caspase activation. In the experiments detailed above, the analysis was restricted to viable cells as identified by propidium iodide staining. To investigate whether the induction of apoptosis was responsible for the reduction in MICA expression, spheroids were assessed after culture with the p53 inhibitor pifithrin alpha or the caspase inhibitor z-vad (Figure S3C in Supplementary Material). Treatment with these inhibitors did not prevent the downregulation of MICA expression observed on spheroids. This suggests that the downregulation of MICA expression on spheroids is not due to the induction of apoptosis.

Treatment of cells with the metalloprotease inhibitor GM6001 is known to increase MICA expression at the cell surface (22). To test if the downregulation of MICA observed in spheroids was due to metalloprotease shedding of MICA, spheroids and confluent adherent cells were treated with the broad spectrum metalloprotease inhibitor GM6001 during culture. GM6001 had no effect on surface MICA expression of spheroids (Figure S3D in Supplementary Material) suggesting that the observed reduction in MICA was not due to metalloprotease action.

### Cell–Cell Contact Downregulates MICA Expression

**Figure 3:**
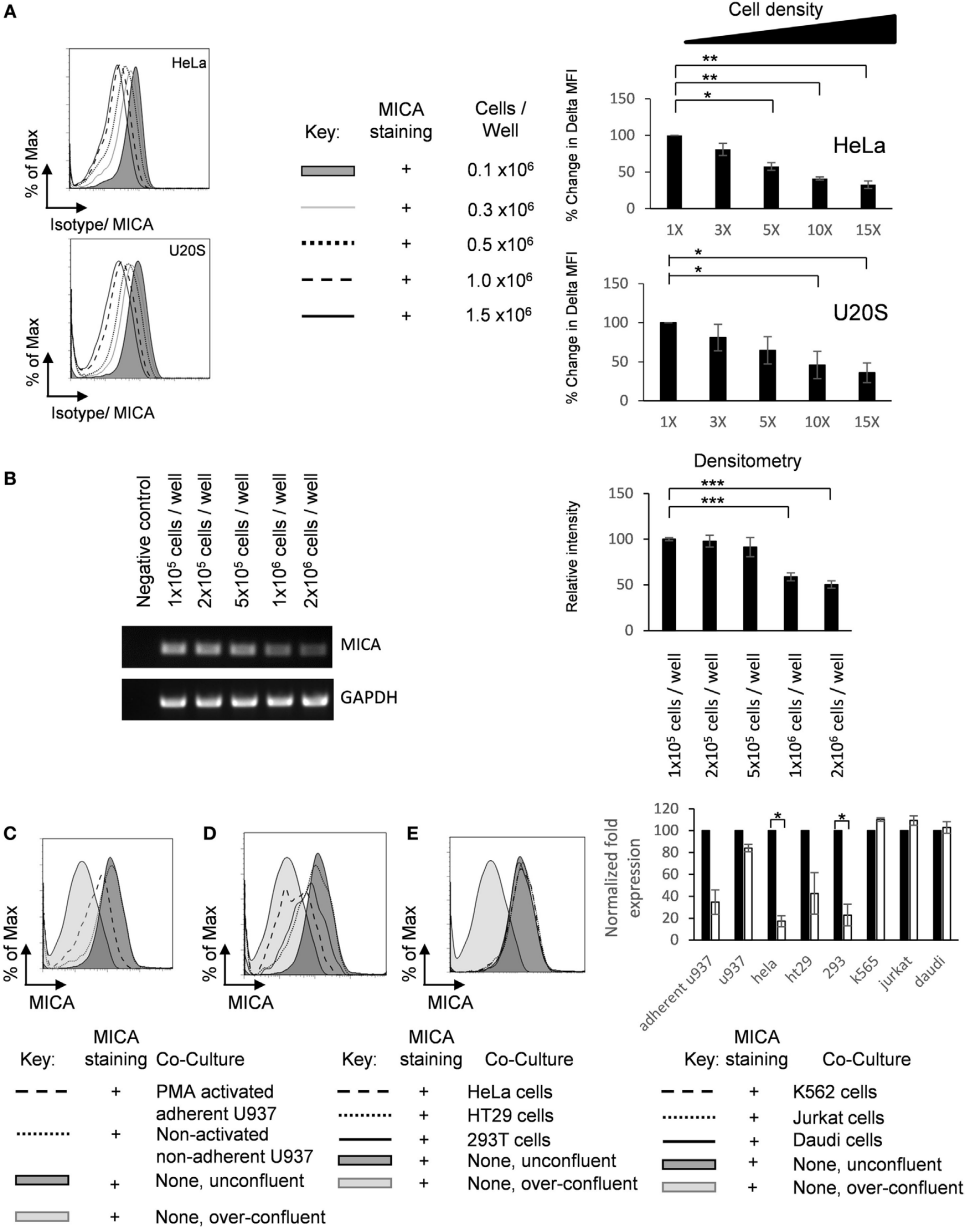
**Adherent cell–cell contact downregulates MICA expression**. **(A)** HeLa and U2OS cells were seeded into six-well plate at indicated cell density, cultured for 72 h, and analyzed for surface MICA expression by flow cytometry *n* = 3. **(B)** U2OS cells were plated at increasing densities and MICA mRNA analyzed compared to a GAPDH control. Normalized quantification is shown in the bar chart. *n* = 3. **(C)** U2OS cells were stained with CFSE and plated at 10% confluence (shaded dark gray histogram), over-confluent (shaded light gray histogram), or cocultured with either PMA-activated adherent U937 cells (dashed histogram) or non-adherent U937 cells (dotted histogram). **(D)** These cells were also grown in coculture with adherent HeLa cells (dashed histogram), adherent HT29 cells (dotted black histogram), or adherent HEK293T cells (solid histogram). **(E)** U2OS cells were also grown with suspension cells K-562 cells (dashed histogram), Jurkat cells (dotted histogram), or Daudi cells (solid histogram). Forty-eight hours later, CFSE-stained U2OS cells were analyzed for MICA expression. *n* = 3 independent experiments.

It has previously been reported that MICA is downregulated on normal fibroblasts upon cell contact (23). We were interested to investigate whether this would also be the case in tumor cell lines and what would be the mechanism for this regulation. Spheroids differ from adherent monolayers not only in their loss of cell–matrix interactions but also in their increased cell–cell interactions. Adherent cells were cultured at increasing cell density, and MICA expression was measured by flow cytometry (Figure 3A). Additionally, cell density regulated MICA mRNA expression was also demonstrated by semiquantitative RT-PCR (Figure 3B).

Furthermore, we wished to investigate whether this reduction of MICA surface expression was due to increased adherent cell–cell contact or was an indirect consequence of the addition of more cells, for example, by reducing the availability of nutrients. Therefore, CFSE-labeled U2OS cells were cocultured under non-confluent (10%) conditions with non-adherent, non-activated U937 monocytes or with U937 monocytes that had been activated with 20 nM PMA for 24 h to render them adherent. Coculture with activated adherent U937 monocytes reduced MICA expression on U2OS cells, while coculture with non-activated non-adherent monocytes had no effect on MICA expression (Figure 3C). Furthermore, coculture with adherent HeLa, HT29, and HEK293T cells also reduced MICA expression (Figure 3D), while culture with non-adherent DAUDI, K-562, and Jurkat cells had no effect on MICA expression (Figure 3E). Together, these results indicate that increased cell–cell contact or cell density of adherent cells is correlated with reduced MICA surface expression on a range of cancer cell lines and indicates that the reduction in MICA expression observed at high cell densities is not simply due to factors such as the reduced availability of nutrients. Therefore, we conclude that, in addition to causing loss of adherence, cell–cell contact also inhibits MICA expression.

### MICA Expression Is Influenced by Extracellular Calcium

**Figure 4:**
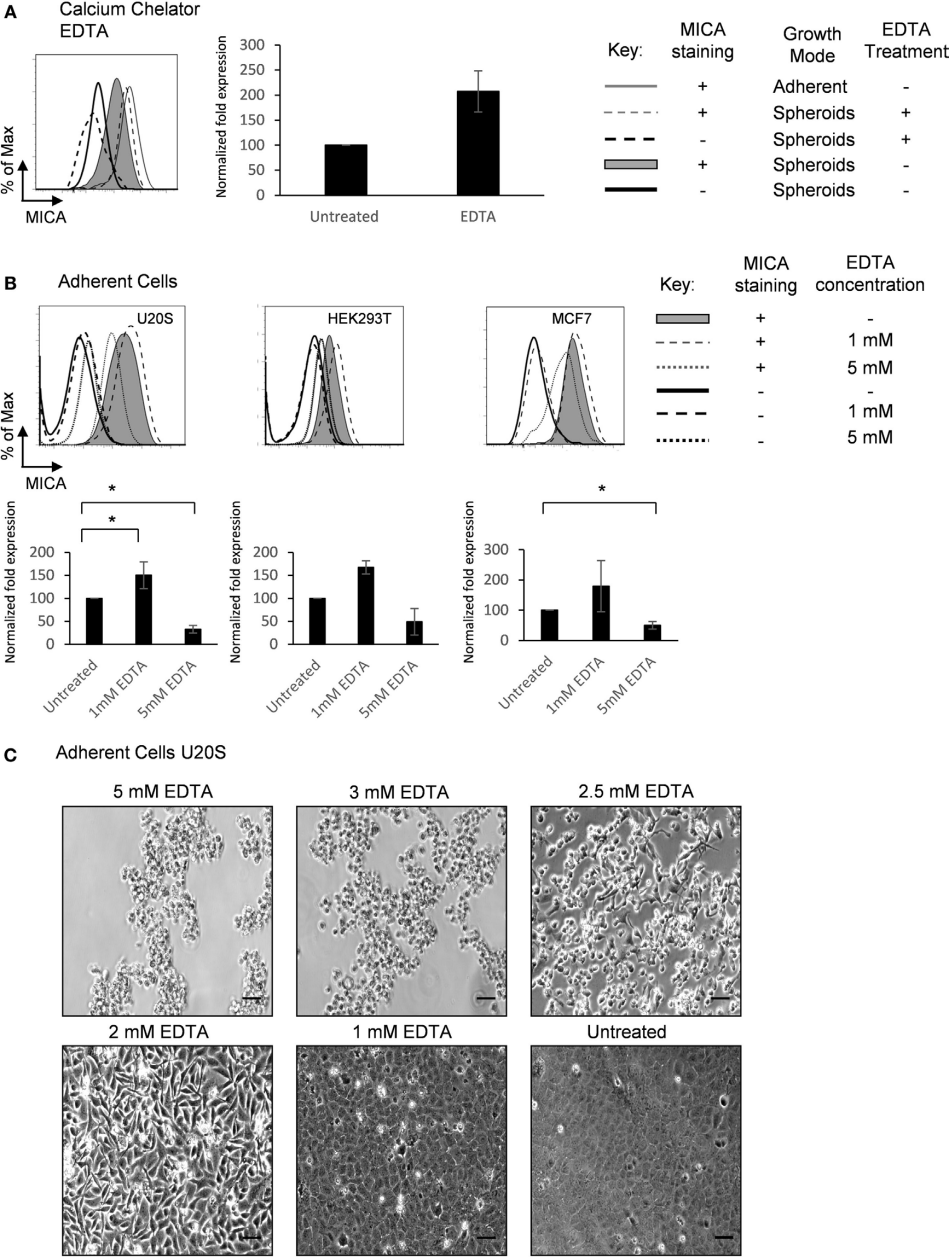
**Extracellular calcium availability modulates MICA surface expression**. **(A)** U2OS cells were cultured either adherently (gray line) or in suspension as spheroids in the presence (dashed gray line; isotype control—dashed black line) or absence of 5 mM EDTA (shaded gray histogram; isotype control—black line) and MICA surface expression analyzed. *n* = 4. **(B)** Adherent U2OS, HEK293T, and MCF7 cells were either left untreated (shaded gray histogram; isotype control—black line) or treated with 1 mM (dashed gray line; isotype control—dashed black line) or 5 mM EDTA (dotted gray line; isotype control—dotted black line) and MICA surface expression analyzed. *n* = 4. **(C)** Images of adherent U2OS cells treated with 5, 3, 2.5, 2, and 1 mM EDTA for 48 h or left untreated. Bar represents 50 µm. *n* = 3.

Calcium-dependent cellular adhesion molecules are essential to cell contact mechanisms (24). To investigate the nature of the cell–cell interactions that regulate MICA expression, spheroids were treated with the calcium chelating agent EDTA. Treatment with up to 5 mM EDTA increased MICA expression on spheroids (Figure 4A). Interestingly, we observed a concentration-dependent adhesion and contact regulation of MICA expression in adherent cells (Figures 4B,C). At 5 mM EDTA, cells detached from the surface and MICA levels were reduced. At the lower concentration of 1–2 mM EDTA treatment, where calcium-dependent cell–cell contact was partially disrupted but cells remained attached to the surface, we observed an increase in the levels of MICA expression. In addition, the more confluent cells are, the less contact area each cell can have with the underlying surface (see Figure 4C untreated vs. 2mM EDTA). Overall, these findings suggest that calcium-dependent cell–matrix adherence promotes MICA expression, while calcium-dependent cell–cell contact inhibits MICA expression.

### MICA Protein Expression Levels Are Reduced after Confluency and Forced Suspension Correlating with FAK Activation

**Figure 5:**
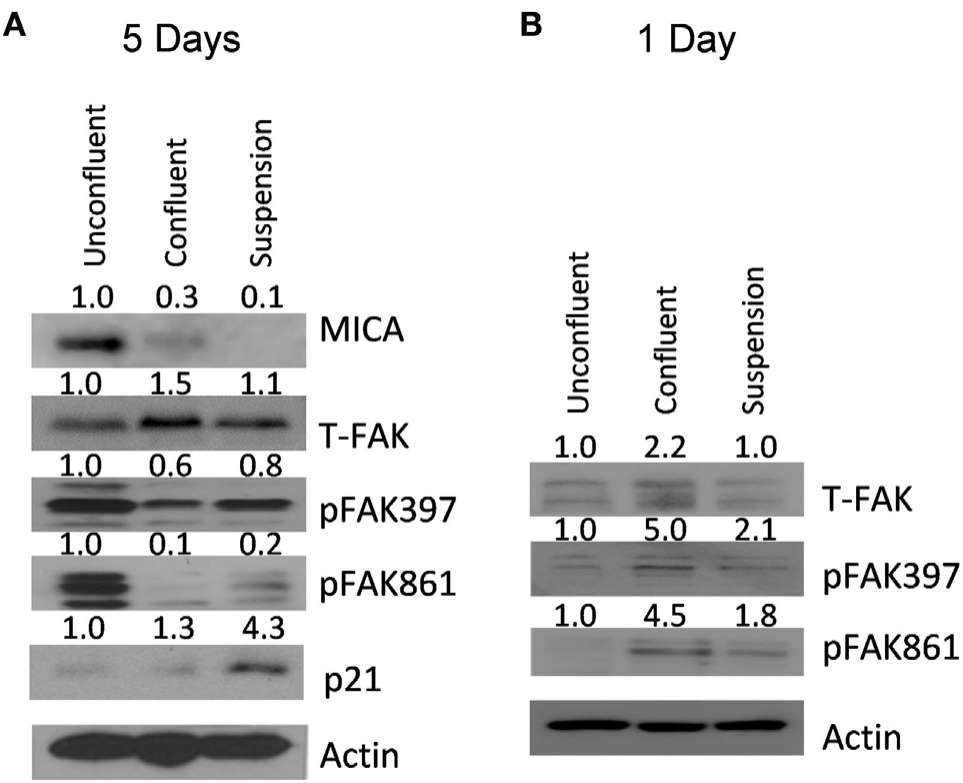
**MICA protein expression levels are reduced after confluency and forced suspension correlating with focal adhesion kinase (FAK) activation**. **(A)** U2OS cells were either grown subconfluently, confluently or as spheroids for 5 days and MICA, p21, beta-actin, FAK, and FAK tyr 397 and tyr 861 phosphorylation measured by western blot. **(B)** U2OS cells were either grown subconfluently, confluently or as spheroids for 1 day and beta-actin, FAK, and FAK tyr 397 and tyr 861 phosphorylation measured by western blot.

Focal adhesion kinase and Src have been shown to be essential for cell survival following loss of surface attachment (25). To test if FAK was activated after loss of adhesion or increased cell contact, U2OS cells were grown either un-confluent, over-confluent or under force suspension for 5 days and cell extracts taken. At day 5, MICA levels were clearly downregulated both at high confluency and at forced suspension (Figure 5A). However, at this time point, FAK phosphorylation levels were clearly reduced. In contrast, p21 levels were found increased in confluent and forced suspension treatments. We therefore elected to look at earlier timepoints. At 1 day after confluency and forced suspension, we observed that FAK phosphorylation levels were indeed increased (Figure 5B).

### FAK/Src Downregulates MICA Expression

**Figure 6:**
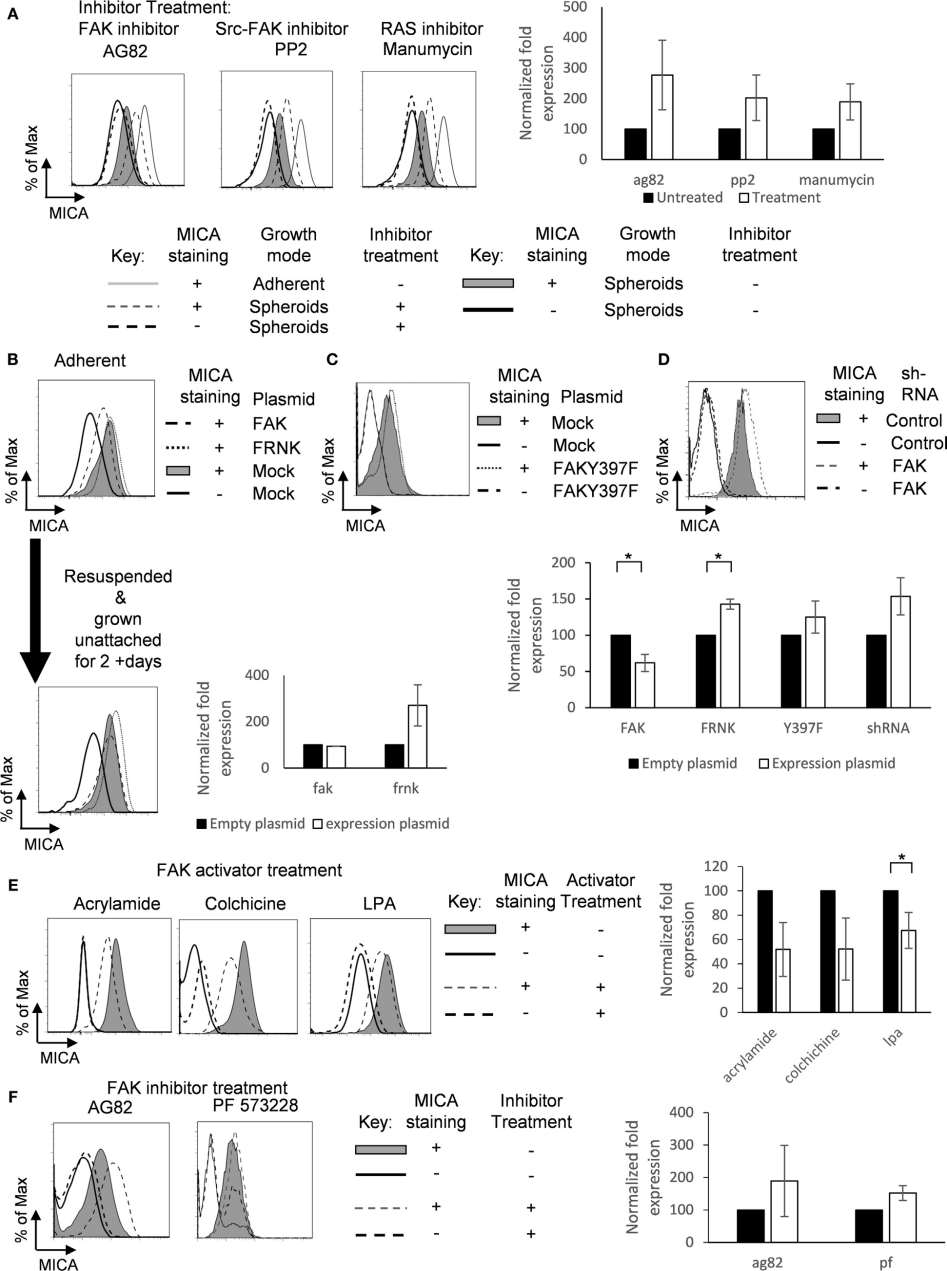
**Focal adhesion kinase (FAK)/Src downregulates MICA surface expression**. **(A)** U2OS cells were cultured as either untreated adherent cells (gray line) or as spheroids and treated with (dashed gray line; isotype control staining—dashed black line) or without (shaded gray histogram; isotype control staining—black line) the FAK inhibitors AG82 and PP2 or manumycin. *n* = 3. **(B)** U2OS cells were transfected with FAK (dashed black line), FRNK (dotted gray line), or with empty vector (full shaded gray histogram; isotype control—black line) for 48 h and then analyzed for MICA expression. Some of these cells were then forced to grow in suspension as spheroids. After 2 days, cells were analyzed for MICA expression. *n* = 3. **(C)** Adherent U2OS cells were either transfected with FAKY397F (dotted gray line) or with empty vector (shaded gray histogram; isotype control staining—black line) and stained for MICA expression. **(D)** U2OS cells were also transfected with either scramble control shRNA (shaded gray histogram; isotype control—black line) or shRNA targeting FAK (dashed gray line; isotype control—dashed black line). **(E)** Adherent U2OS cells were left untreated (shaded gray histogram; isotype control—black line) or treated with the FAK activators acrylamide, colchicine, or lysophosphatidic acid and stained for MICA surface expression. **(F)** Adherent U2OS cells were left untreated (shaded grey histogram; isotype control—black line) or with the FAK inhibitors tyrphostin AG82 or PF 573228 and stained for MICA expression (dashed grey line; isotype control staining—dashed black line). *n* = 3 independent experiments.

We then sought to determine whether modulating FAK activation would regulate MICA surface expression. Treatment of spheroids with the FAK/Src inhibitors tyrphostin AG82 or PP2 increased MICA expression (Figure 6A). MICA expression was also increased by treatment of spheroids with manumycin, an inhibitor of Ras, which is downstream of FAK (Figure 6A). We then investigated whether FAK expression could influence the surface expression of MICA. Overexpression of FRNK, a dominant negative FAK mutant, had only a small effect on MICA expression in non-confluent adherent cells, but overexpression of FAK reduced surface levels of MICA (Figure 6B). Transfected cells were also resuspended over agarose for 2 days, and then MICA expression was analyzed. Here, we could see that the FRNK transfected cells had higher MICA expression compared to mock-treated suspension cells. These results indicated that inhibition of FAK/Src signaling leads to increased levels of MICA in spheroids, suggesting that FAK/Src can play a role in regulating MICA expression. Adherent cells transfected with the kinase deficient mutant FAKY397F also showed a slight upregulation in MICA expression (Figure 6C). Additionally, knockdown of FAK with shRNA led to upregulation of MICA expression compared to control scramble shRNA (Figure 6D). FAK and shRNA expression was monitored through EGFP expression on an IRES of the plasmids. To further investigate whether FAK activation could reduce MICA expression on adherent cells, cells were cultured with the FAK activators acrylamide (26), colchicine, and lysophosphatidic acid (LPA) (27). MICA expression was reduced following treatments with these FAK activators (Figure 6E). Treatment of adherent cells with the FAK/Src inhibitor tyrphostin AG82 and PF 573228 upregulated MICA surface expression (Figure 6F). Together, these data show that FAK/Src can downregulate MICA expression in adherent cells.

### Cellular Growth Mode Influences NK Cell-Mediated Cytotoxicity

**Figure 7:**
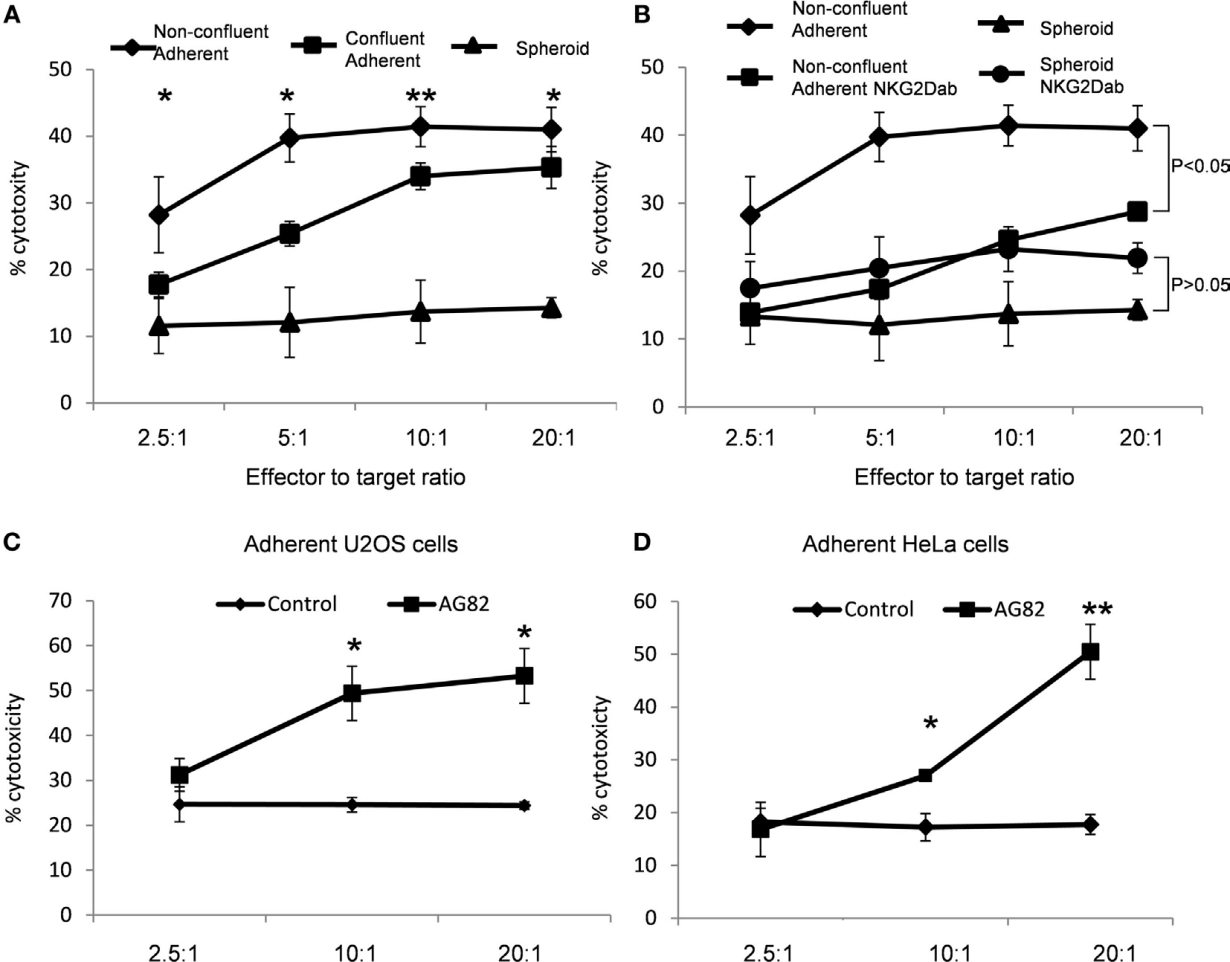
**MICA expression correlates with increased natural killer (NK) cell-mediated cytotoxicity**. **(A)** U2OS cells were either plated to grow as non-confluent adherent cells, as confluent adherent cells or were cultured in suspension. After 5 days, cells were exposed to NK cells in a chromium-release assay. **(B)** Non-confluent cells and spheroids were also subjected to NK cells pretreated with a blocking NKG2D antibody. **(C)** Adherent U2OS and **(D)** HeLa cells were either mock-treated or treated with the focal adhesion kinase inhibitor AG82 prior to exposure to NK cells in a chromium-release assay. Points plotted represent mean of four replicates. Error bars show SEMs. To test for significance, a Student’s *t*-test was performed with the degree of cytotoxicity values of each ratio against the other conditions. *p < 0.05, **p < 0.01.

Next, we tested whether the different levels of MICA expression observed on spheroids, confluent adherent cells, and non-confluent adherent cells were functionally relevant and whether they influenced susceptibility to NK cell-mediated killing. Spheroids, confluent, and non-confluent adherent cells were incubated with NK cells in a chromium-release killing assay. The non-confluent adherent cells with the highest MICA expression were more susceptible to NK cell killing than confluent adherent cells. Suspension spheroids were killed less readily than the confluent adherent cells (Figure 7A). Treatment with anti-NKG2D antibody significantly reduced the susceptibility of non-confluent cells to NK cell-mediated killing (Figure 7B). In line with the observed reduction in MICA expression, treatment with the NKG2D blocking antibody had no significant impact on spheroid susceptibility. MICA expression therefore correlates with susceptibility to NK cell killing.

To confirm whether NK cell susceptibility was modulated by FAK/Src activity, adherent cells were treated with the FAK/Src inhibitor AG82 to increase MICA expression and these cells were then exposed to NK cells. AG82-treated U2OS and HeLa cells were more susceptible to NK cell-mediated killing than mock-treated cells (Figures 7C,D). Therefore, FAK/Src signaling inhibition results in increased susceptibility to NK cell killing.

### *In Vivo* MICA Expression of Human in Mice Xenograft

**Figure 8:**
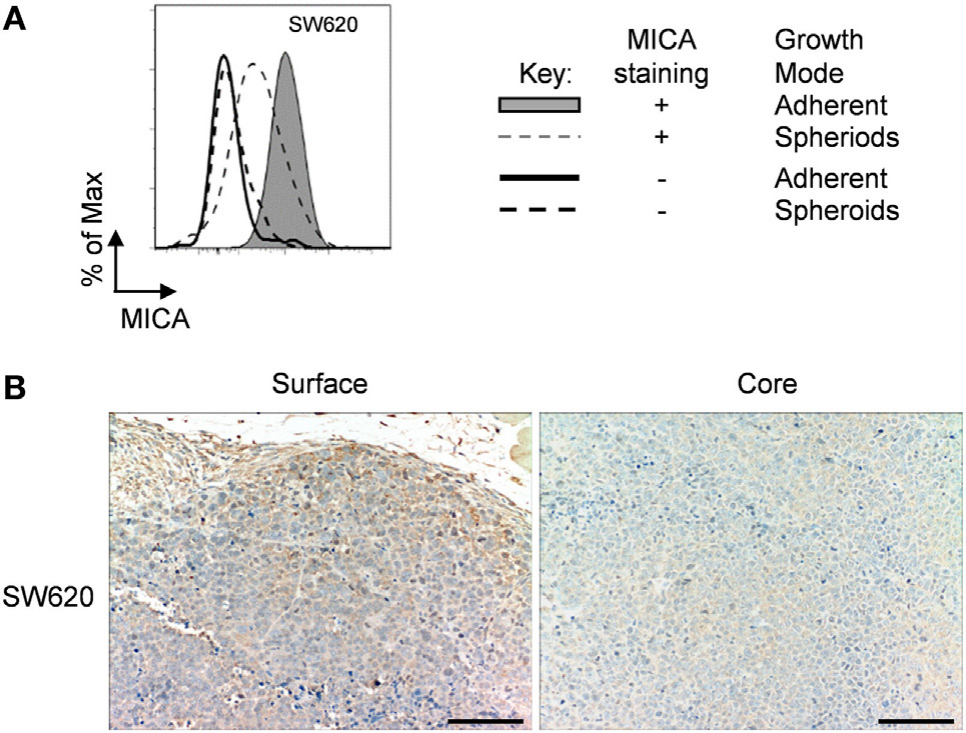
**Immunohistochemistry of SW620 xenograft**. **(A)** Adherent SW620 cells were cultured over agarose-coated plates for 5 days to force formation of non-adherent spheroids (dashed gray line) or were cultured under non-confluent adherent conditions (shaded gray histogram) and stained for MICA surface expression (isotype control—black line for adherent cells or dashed black line for non-adherent spheroid suspension cells). **(B)** Immunohistochemistry images of an SW620 xenograft stained for MICA expression showing the surface and the core of the tumor. Bar represents 50 µm.

All previous experiments were done *in vitro* with well-established cell lines, but we were interested to see if what we saw *in vitro* could be translated *in vivo*. For this, we selected a cell line, SW620 adenocarcinoma, derived from a metastatic lymph node tumor (28). We first confirmed that this cell line could also downregulate MICA expression after loss of adhesion (Figure 8A). We then stained an SW620 in nude mice xenograft for MICA expression. Here, we could see that the surface of the tumor had a stronger MICA expression than at the core (Figure 8B), recapitulating the *in vitro* results. Therefore, our observation may be especially important, as treatments that upregulate MICA expression in spheroids may upregulate MICA expression *in vivo* where tumor MICA expression is often negligible.

## Discussion

MICA is an important ligand for the immune receptor NKG2D, which plays an activating role in NK and T cells. This study demonstrates that MICA expression is strongly influenced by cell-surface adhesion and cell–cell contact and this has implications for understanding the immunopathogenesis of cancer metastasis.

We initially observed that MICA was expressed on almost all adherent cell lines, but expression was low or absent on cell lines which normally grow in suspension. One of the MICA expressing suspension cell lines was K-562, which expresses the BCR-ABL fusion oncoprotein which has been reported to induce MICA expression directly (29). MICB expression in contrast was less often found on adherent cell lines and on more suspension cell lines like HL-60. ULBP2 was found in almost all cell lines regardless of whether they were adherent or not. While adherent cells were shown to express high levels of MICA, MICA levels were considerably reduced on cells that had detached from the surface under the same culture conditions. Forcing cells to grow in suspension as spheroids through culture over agarose led to a downregulation of MICA expression.

Focal adhesion kinase and Src play key roles in cell–cell contact and have been shown to be essential for growth in suspension by inhibition of anoikis, which is death resulting from loss of matrix attachment (25, 30). We investigated whether the FAK/Src signaling axis was involved in regulating MICA expression. MICA expression in spheroids was increased by chemical inhibition of FAK/Src or Ras and by overexpression of FRNK or FAKY397F dominant negative FAK mutants. In adherent cells, chemical activation and overexpression of FAK downregulated MICA expression, while the opposite effect was observed following with chemical inhibition of FAK and with shRNA targeted against FAK. Consistent with our observations, TGF-β has been shown to activate FAK and to downregulate MICA expression (22, 31). These data indicate that activation of the FAK/Src pathway has a negative regulatory influence on MICA expression.

Spheroids have high levels of cell–cell contact because they are in a three-dimensional arrangement with other cells. We found that increased cell–cell contact reduced MICA expression on cancer cell lines and that signaling through the FAK/Src axis plays a key role in this. These findings provide a mechanistic foundation for understanding earlier observations in untransformed adherent primary cells (23). Our results show that loss of cell–cell contact following 1 mM EDTA treatment upregulated MICA expression, but once EDTA levels were high enough to inhibit surface adhesion (5 mM) MICA levels fell to even lower levels than on untreated cells. This may be due to the observation that at lower EDTA concentrations, cells lose cell–cell contact by blocking cadherin adhesive binding activity (32) and have more surfaces to adhere. At higher EDTA levels, integrin signaling is completely blocked (33), although if FAK is not activated, cells would die through anoikis (25). Thus, the effect of loss of surface adhesion is dominant over that of cell–cell contact.

The reduced MICA expression observed on spheroids compared to non-adherent cells is functionally significant, and spheroids are less susceptible to NK cell cytotoxicity. Tumor spheroids are known to be less susceptible to cytotoxic cells, and this has been attributed to reduced antigen presentation associated with downregulation of HSP-70 (3). Transfection of HSP-70 partially restored susceptibility to cytotoxic attack. This effect may in part be due to an upregulation of NKG2D ligands, as HSP-70 transfection has been reported to induce MICA overexpression (34). Here, we could observe that most NKG2D ligands were downregulated following forced suspension. MICA is the NKG2D ligand most highly expressed on many adherent cell lines, and this was significantly downregulated. Although MHC class I expression was also downregulated, this did not make the cells more sensitive; therefore, the NKG2D ligand downregulation appears to be dominant. The MHC class I downregulation may also have an important effect on T cell recognition of metastatic cells.

As MICA and MICB are not expressed in mice (35), many regulatory studies have focused on *in vitro* experiments. To investigate the *in vivo* relevance of our *in vitro* results, we analyzed MICA expression in a tumor xenograft. Here, we could observe that MICA expression was higher on cells at the surface of the tumor than in the core. The immunohistochemistry images showed that cells on the surface of the tumor were more loosely packed, analogous to the non-confluent adherent cells we studied *in vitro*. The core, in contrast, was compact showing more contact and less space between the cells, comparable to over-confluent cells. Therefore, the MICA staining of an *in vivo* tumor correlated with the *in vitro* findings in this study.

The physiological condition where “loss of adhesion” could take place is during tumor cell extravasation. The relationship between MICA expression and metastasis had been observed with uveal melanoma where metastatic melanoma cells have lower MICA expression than primary tumors (36). The failure to induce cell-surface MICA expression upon loss of surface adhesion, thereby facilitating immune evasion, could be a significant step in the development of metastatic cancer. Therefore, in a future study it would be interesting to analyze MICA expression on tumor circulating cells from a range of MICA positive tumors and test if these cells lose MICA expression *in vivo* as a mechanism of immune evasion.

In summary, this study shows that spheroids and confluent adherent cells downregulate MICA expression in a FAK/Src-dependent manner. We have identified a mechanism whereby adhesion and cell contact can modulate the extent to which a cell can be recognized by cytotoxic cells expressing NKG2D. Targeting this pathway may be of therapeutic value in reducing the probability of tumor immune evasion and metastasis.

## Materials and Methods

### Cell Culture

The cell lines 786-O, A549, BS125, primary human fibroblasts, HEK293T, HeLa, Hep3b, HEPG2, HT-1080, HT29, HUH7, MCF7, MO59K, RCC4, SF767, SKOV3, SUSA, SW480, SW620, T47D, U2OS, U343MG, U373MG, U87MG, and ZR751 were cultured in D10: DMEM (Sigma, St. Louis, MO, USA) supplemented with 10% fetal calf serum (FCS), 50 U/ml penicillin, 50 µg/ml streptomycin, and 4mM l-glutamine. Normal human astrocytes were grown in Astrocyte Medium (ScienceCell) containing astrocyte growth supplement and FCS. BCL-B7, C1R, DAUDI, HL-60, HUT-78, Jurkat, K-562, NK92, NKL, RAJI, THP-1, U937, and YT were cultured in RPMI-1640 with 10% FCS, 50 U/ml of penicillin, and 50 µg/ml of streptomycin (Sigma). NK92 and NKL cell lines were supplemented with IL-2 (500 U/ml) and 1 mM sodium pyruvate. Heparinized blood obtained from healthy donors was collected and centrifuged at 400 × *g* for 20 min over Ficoll-Paque (Sigma, St. Louis, MO, USA). PBMC were collected at the interface, washed twice with DMEM, and analyzed immediately, without culture. To promote growth as spheroids, 24-well plates were coated with 1% agarose in PBS. The wells were then washed twice in full media prior to the addition of these cells.

### Inhibition and Activation Treatments

The following inhibitors or activators were used for signaling studies: 1–5 mM EDTA, 10 µM PP2, 1 µM colchicine (Sigma), 6 mM acrylamide (National Diagnostics, Atlanta, GA, USA), 1 µM AG82 FAK/Src inhibitor, 3 µM manumycin (Calbiochem, San Diego, CA, USA), 500nM PF 573228 (R&D Systems, MN, USA), and 5 µM LPA (Enzo Life Sciences).

### Transfection

Cells were transfected with FAK, FAKY397F, FRNK (gifts from C. Chen, University of Pennsylvania), shFAK (GATCCCCGAAGTTGGGTTGT CTAGAATTCAAGAGATTCTA GACAACCCAACTTCTTTTTA), and shScramble in pSIREN (Clontech) using the lipofectamine transfection system per the manufacturer’s manual (Invitrogen, Carlsbad, CA, USA).

### CFSE Labeling

A total of 1 × 10^6^ cells were pelleted, and 10 µM CFSE staining solution was added directly to the cells, which were gently resuspended and incubated at 37°C for 20 min. Complete medium was then added to stop the reaction. Finally, cells were washed twice with PBS.

### Flow Cytometry

For the analysis of NKG2D ligand expression, cells were washed twice and incubated with 5mM EDTA in PBS for 5 min to detach the cells. Spheroids were also treated with 5mM EDTA and manually disrupted to achieve a single cell suspension. Cells were then resuspended in PBS supplemented with 0.03% azide with 5% BSA for blocking. Staining was performed in the same buffer using monoclonal antibodies against MICA (2C10, Santa Cruz, Santa Cruz, CA, USA), MICB (mAb 1599, R&D System, Minneapolis), ULBP1 (mAB 170818, R&D System, Minneapolis), ULBP2 (MAB1298, R&D System, Minneapolis), ULBP3 (Clone 166510, R&D System, Minneapolis), HLA-A, B, C (W6/32, Thermo Fisher, UK), or IgG1, isotype control monoclonal antibodies (eBiosciences, San Diego, CA, USA). Cells were then washed twice and labeled with FITC-labeled polyclonal rabbit anti-mouse IgG (STAR9B, Serotec, Raleigh, NC, USA) or goat anti-mouse IgG Cy5 conjugated (AP124S, Chemicon). Propidium iodide (Sigma) was added at a concentration of 50 µg/ml to identify viable cells. Flow cytometry was performed using a BD^®^FACSCanto machine and FACSDiva Software (BD Biosciences, San Jose, CA, USA) and analyzed using FlowJo software (Tree Star).

### Chromium-Based Killing Assay

A total of 1 × 10^6^ target cells were dissociated from plates, washed twice in RPMI, resuspended in 100 µl of a 7.4 MBq ${\text{Na}}_{2}^{51}{\text{CrO}}_{4}$ solution (GE Healthcare Life Sciences, Piscataway, NJ, USA), diluted in growth media, and then incubated for 1 h in a humidified 5% CO_2_ atmosphere at 37°C. After washing three times with RPMI, the labeled cells were resuspended in their growth media and seeded in 96-well plates at 5 × 10^4^ cells per well in quadruplicate. Effector cells were added at the specified effector to target cell ratios (E:T) and were incubated for 4 h at 37°C, 5% CO_2_. The supernatant was removed, added to Optiphase Supermix (PerkinElmer), and radioactivity measured with a beta counter (MicroBeta TriLux, PerkinElmer, Waltham, MA, USA). The degree of cytotoxicity was determined according to the formula: % cytotoxicity = [(sample release) − (spontaneous release)]/[(maximum release) − (spontaneous release)]. Spontaneous release was determined by incubating target cells in medium alone. Maximum release of target cells was measured following treatment with 10% detergent Triton X-100 (Sigma). Error bars represent the SD error of the acquired values. To test for significance, a Student’s *t*-test was performed with the degree of cytotoxicity values of each ratio against the other conditions. *p < 0.05, **p < 0.01.

### Semiquantitative PCR

Total RNA was extracted using TRIzol Plus kit (Invitrogen). One microgram of RNA was reverse transcribed into cDNA using OligodT (Invitrogen), and BioScript reverse transcriptase (Bioline). Twenty nanograms of cDNA were subject to semiquantitative PCR reaction using BioTaq polymerase (Bioline) for 28 cycles for MICA and 22 cycles for GAPDH. The PCR primers are MICA forward TCTTCCTGCTTCTGGCTGGCAT; MICA reverse GGGTCATCCTGAGGTCCTTTCCG; GAPDH forward TCCATGACAACTTTGGTATCGTGG; and GAPDH reverse CACCACCCTGTTGCTGTAGCC. PCR reactions were separated by 1% agarose gel and quantified by densitometry using ImageJ software (NIH).

### Light Microscopy

Cells were plated in a 12-well plate and treated for 48 h with increasing concentrations of EDTA. Cells were then imaged with a Nikon Eclipse TE2000U fluorescence inverted microscope using a 20× objective and Hamamatsu B&W C4742-95 Orca hi-sensitivity CCD camera using IPLab imaging software.

### Western Blotting

Cells were homogenized in lysis buffer (50 mM Tris–HCl pH 7.5, 120 mM NaCl, 1% NP-40, 40 mM β-glycerophosphate, 1 mM benzamidine, 1 mM phenylmethylsulfonyl fluoride, 1 mM sodium orthovanadate, 25 mM NaF, and 2 µM microcystin-LR). After centrifugation at 15,000 × *g* for 15 min at 4°C, supernatants were collected and soluble protein concentrations were determined using the Bradford assay (Bio-Rad, Hercules, CA, USA). Fifty micrograms of protein extracts were separated on 8–12% SDS-PAGE and transferred onto PVDF membrane (Millipore, Bedford, MA, USA) by electroblotting. Membranes were blocked with 5% BSA in TBST (50 mM Tris–HCl pH 7.5, 150 mM NaCl, 0.1% Tween 20) and incubated overnight with (1:100 v:v dilution) primary antibody against MICA (2C10), total FAK (C-20), Tyr397 p-FAK, and Tyr 861FAK and beta-actin (Santa Cruz, CA, USA). Subsequently, membranes were incubated with appropriate HRP-linked secondary antibody (1:2,000 v:v dilution) in PBST (anti-mouse HRP, GE Healthcare, and anti-goat HRP, Santa Cruz) for 1 h at room temperature. Blots were analyzed by enhanced chemiluminescence reagents. Quantification of western blots was done through densitometry using ImageJ (NIH) software.

### Immunohistochemistry

IHC was performed using a rabbit polyclonal antibody against MICA (ab62540, Abcam, UK) at 1:30 dilution. For detection, deparaffinized and rehydrated slides were pretreated in 10 mM citrate buffer, pH 6.0, at 98°C for 60 min for renaturation. The detection ABC method was used per the manufacturer’s instructions. MICA specific signals were recorded from slides of xenografts of SW620 cell lines injected in the flank of Hsd nude mice using an automated instrument reagent system (Discovery XT, Ventana Medical System, Inc.) per the user manual. Images of sections were captured (Nikon, YTHM) and analyzed using Image Access Enterprise 7 and ImageJ software. SW620 xenograft slide was a kind gift from Dr. Lei Zhang in Dr. Brian A. Hemmings lab with help from Sandrine Bichet from the Friedrich Miescher Institute for Biomedical Research, Basel, Switzerland. Tissue samples were processed as described previously (37) with the recommendations of the Ethical Committee of the University Hospital of Basel. The protocol was approved by the Ethical Committee of the University Hospital of Basel.

### Statistical Analysis

As shown on each figure, *n* indicates the number of experiments using cells from independent experiments. Where three or more experiments were conducted a two-tailed Student’s *t*-test with two samples unequal variance type was conducted. Means were considered significantly different when *p < 0.05, **p < 0.01. Error bars represent the SD of the mean.

## Author Contributions

GM and CO’C designed the research; GM, DL, and MM performed the experiments; GM and DL performed the statistical analysis; GM, DL, MM, AW, and CO’C analyzed data and wrote the manuscript.

## Conflict of Interest Statement

The authors declare that the research was conducted in the absence of any commercial or financial relationships that could be construed as a potential conflict of interest.

## Supplementary Material

Figure S1 **Schematic diagram and light microscopic images of non-adherent cells, adherent cells, and spheroids**. Growth mode, schematic diagrams, and light microscope images of cells cultured normally in **(A)** adherence, **(B)** suspension, **(C)** over-confluent with naturally detaching cells, or as **(D)** spheroids showing grade of contact, adherence, and MICA expression. Bar represents 50 µm.

Figure S2 **HLA-A, B, C, and NKG2D ligand expression after forced suspension in HeLa and U2OS cells**. **(A)** Adherent HeLa and **(B)** U2OS cell lines were cultured over agarose-coated plates for 5 days to force formation of non-adherent spheroids (dashed gray line) or were cultured under non-confluent adherent conditions (shaded gray histogram) and stained for MICA, MICB, ULBP1, ULBP2, ULBP3, and MHC class I surface expression (isotype control—black line for adherent cells or dashed black line for non-adherent spheroid suspension cells). *n* = 3.

Figure S3 **MICA expression inhibition is not caused by hypoxia, apoptosis, or metalloproteases**. **(A)** U2OS cells were cultured adherently and under hypoxic conditions (1% oxygen) for 48 h (dotted gray line), under hypoxia for 24 h followed by 24 h oxygen–reoxygenation (dashed black line) or cultured in normoxia (shaded gray histogram) and stained for MICA surface expression. Isotype control—black line. *n* = 3. **(B)** U2OS, HeLa, and 786-O adherent cells were either left untreated (full shaded gray histogram; isotype control—thick black line) or incubated under anoxic (0.1% oxygen) conditions for 48 h (dashed thin black line; isotype control—dashed thick black line). *n* = 3. **(C)** U2OS cells were either cultured adherently (gray line; isotype control—black line) or as spheroids and treated with either pifithrin or z-vad (dashed black line) or mock-treated (shaded gray histogram) for 48 h, before staining for MICA expression. The downregulation of MICA on spheroids compared to adherent cells is not abrogated by these inhibitors. *n* = 2. **(D)** U2OS cells were either cultured adherently (gray line), as spheroids (shaded gray histogram; isotype control—black line) or as spheroids and treated with the metalloprotease inhibitor GM6001 (dashed gray line; isotype control—dashed black line) for 48 h while grown in suspension. *n* = 2.
