# Supplementary figures and images for: MICA Expression Is Regulated by Cell Adhesion and Contact in a FAK/Src-Dependent Manner
Source: Front Immunol. 2017 Jan 19;7:687. doi: 10.3389/fimmu.2016.00687 (PMC5243850; doi:10.3389/fimmu.2016.00687)

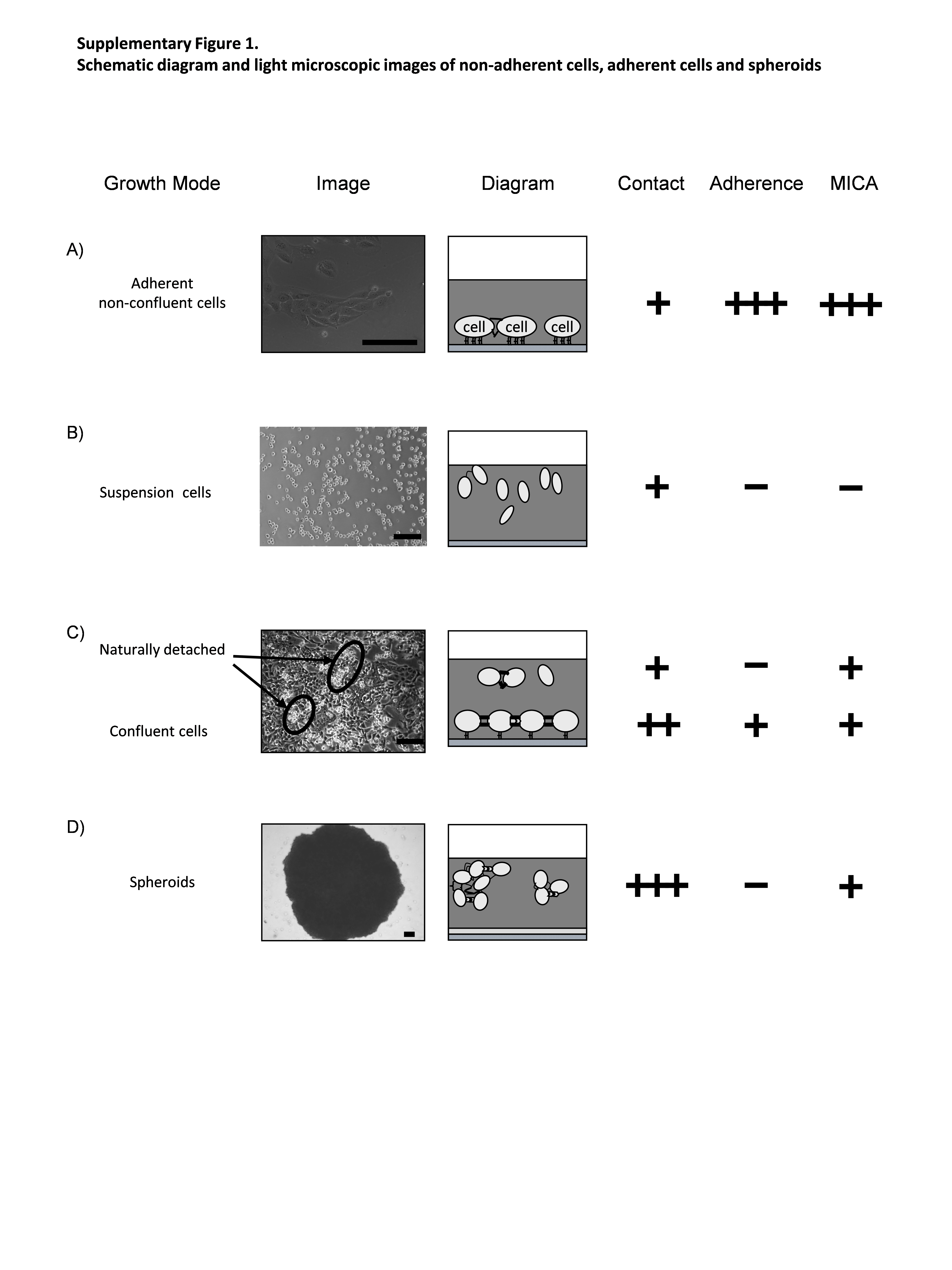

Supplement: Figure S1 — Schematic diagram and light microscopic images of non-adherent cells, adherent cells, and spheroids. Growth mode, schematic diagrams, and light microscope images of cells cultured normally in (A) adherence, (B) suspension, (C) over-confluent with naturally detaching cells, or as (D) spheroids showing grade of contact, adherence, and MICA expression. Bar represents 50 µm. [file image_1.tif]

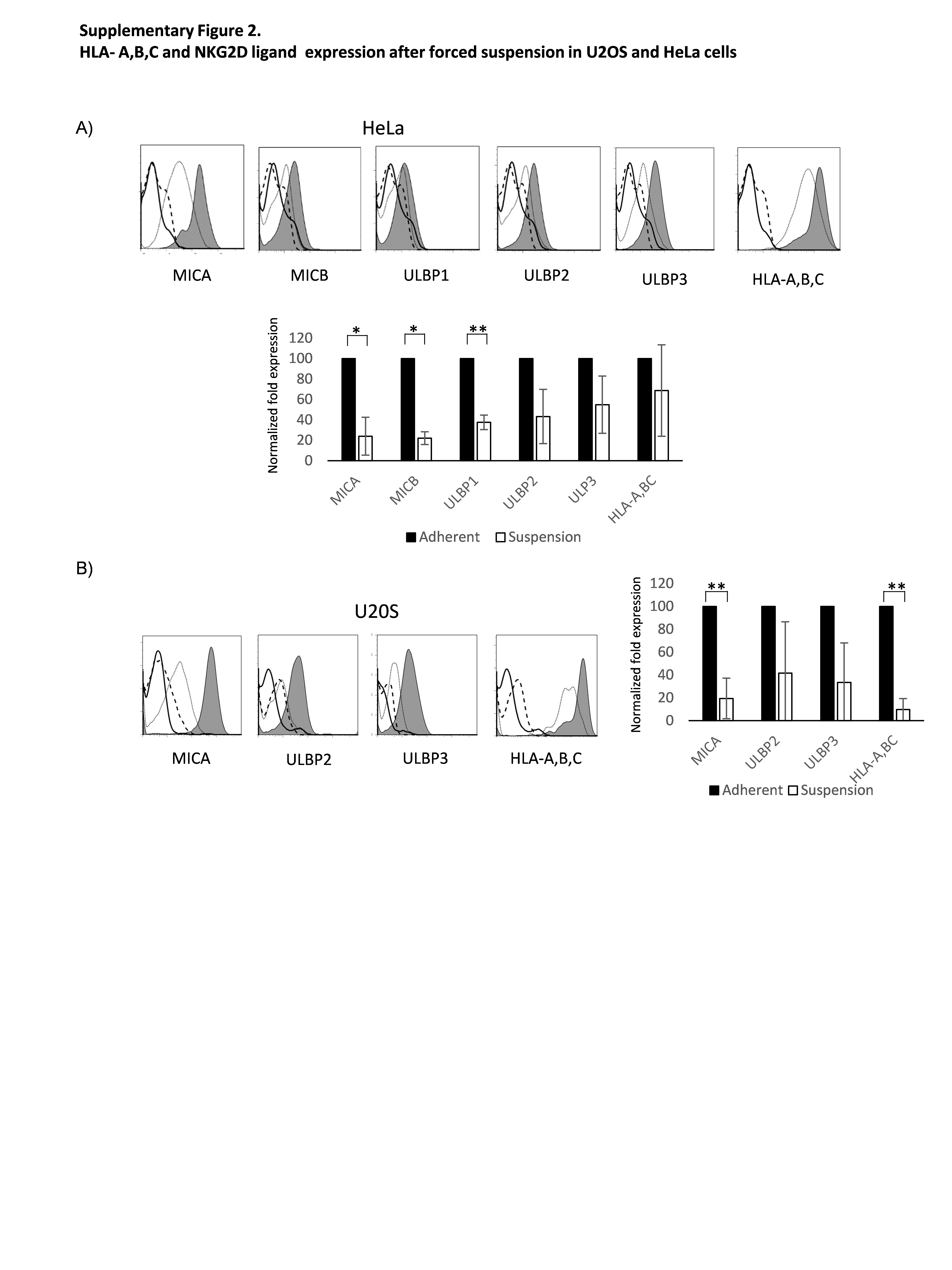

Supplement: Figure S2 — HLA-A, B, C, and NKG2D ligand expression after forced suspension in HeLa and U2OS cells. (A) Adherent HeLa and (B) U2OS cell lines were cultured over agarose-coated plates for 5 days to force formation of non-adherent spheroids (dashed gray line) or were cultured under non-confluent adherent conditions (shaded gray histogram) and stained for MICA, MICB, ULBP1, ULBP2, ULBP3, and MHC class I surface expression (isotype control—black line for adherent cells or dashed black line for non-adherent spheroid suspension cells). n = 3. [file image_2.tif]

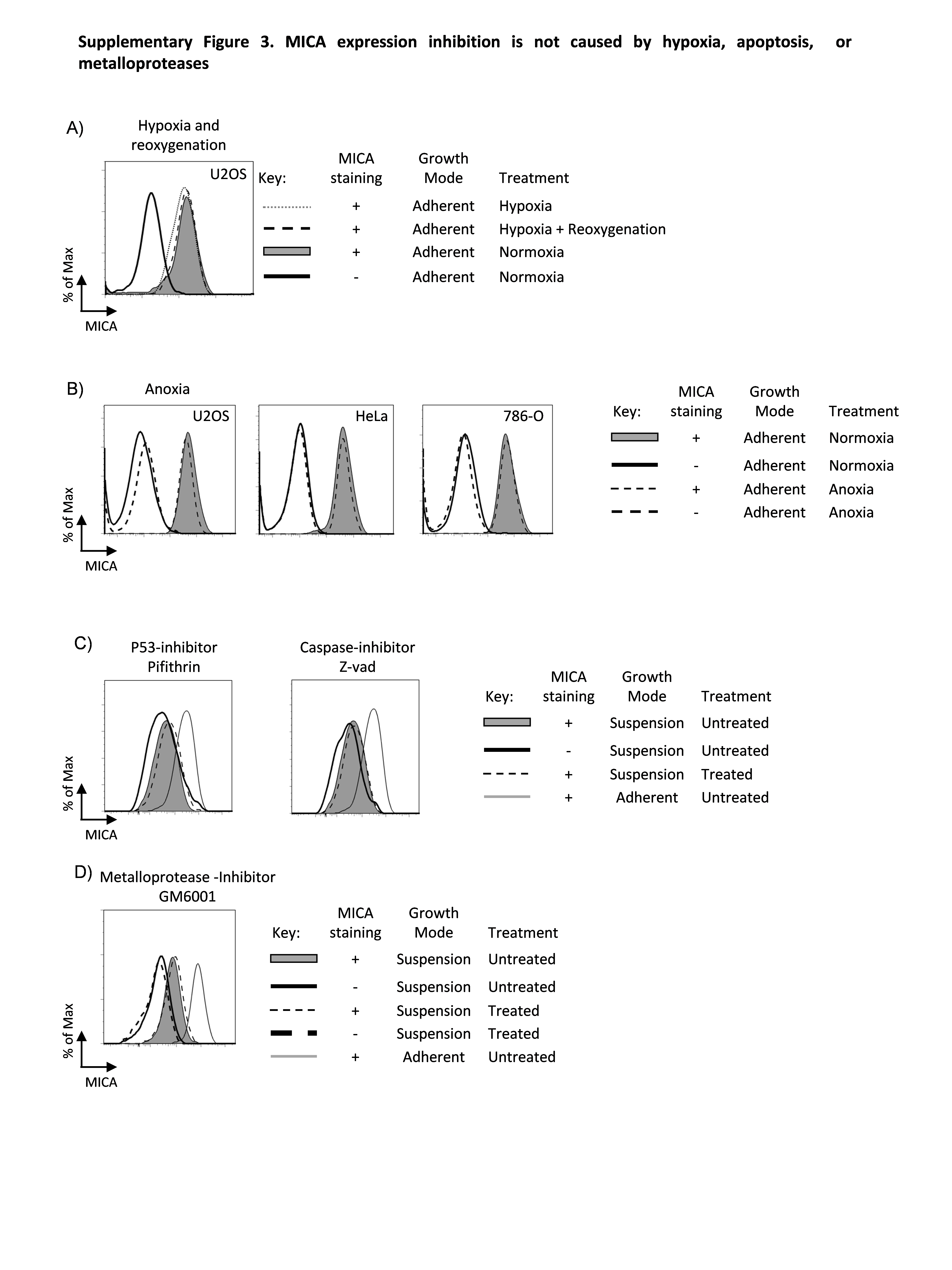

Supplement: Figure S3 — MICA expression inhibition is not caused by hypoxia, apoptosis, or metalloproteases. (A) U2OS cells were cultured adherently and under hypoxic conditions (1% oxygen) for 48 h (dotted gray line), under hypoxia for 24 h followed by 24 h oxygen–reoxygenation (dashed black line) or cultured in normoxia (shaded gray histogram) and stained for MICA surface expression. Isotype control—black line. n = 3. (B) U2OS, HeLa, and 786-O adherent cells were either left untreated (full shaded gray histogram; isotype control—thick black line) or incubated under anoxic (0.1% oxygen) conditions for 48 h (dashed thin black line; isotype control—dashed thick black line). n = 3. (C) U2OS cells were either cultured adherently (gray line; isotype control—black line) or as spheroids and treated with either pifithrin or z-vad (dashed black line) or mock-treated (shaded gray histogram) for 48 h, before staining for MICA expression. The downregulation of MICA on spheroids compared to adherent cells is not abrogated by these inhibitors. n = 2. (D) U2OS cells were either cultured adherently (gray line), as spheroids (shaded gray histogram; isotype control—black line) or as spheroids and treated with the metalloprotease inhibitor GM6001 (dashed gray line; isotype control—dashed black line) for 48 h while grown in suspension. n = 2. [file image_3.tif]
